# Bixin, a New Atheroprotective Carotenoid Candidate, Prevents oxLDL-Induced Cytotoxicity and Mitochondrial Dysfunction in Macrophages: Involvement of the Nrf2 and NF-κB Pathways

**DOI:** 10.3390/foods13132002

**Published:** 2024-06-25

**Authors:** Sabrina Somacal, Luana Caroline Schüler da Silva, Jade de Oliveira, Tatiana Emanuelli, Andreza Fabro de Bem

**Affiliations:** 1 Graduate Program on Pharmacology, Center of Health Sciences, Federal University of Santa Maria, Santa Maria 97105-900, RS, Brazil; sabrina.somacal@ufsm.br; 2 Department of Biochemistry, Federal University of Santa Catarina, Florianopolis 88040-900, SC, Brazil; luanaschuler@gmail.com; 3 Department of Biochemistry, Federal University of Rio Grande do Sul, Porto Alegre 90035-000, RS, Brazil; 00317650@ufrgs.br; 4 Department of Food Technology and Science, Center of Rural Sciences, Federal University of Santa Maria, Santa Maria 97105-900, RS, Brazil; tatiana.emanuelli@ufsm.br; 5 Laboratory of Bioenergetic and Metabolism, Institute of Biological Science, University of Brasília, Brasília 70910-900, DF, Brazil

**Keywords:** foam cells, oxidative stress, inflammation, lycopene, *Bixa orellana*

## Abstract

The accumulation of oxidized low-density lipoprotein (oxLDL) and its toxicity in the arterial wall have been implicated in atherosclerosis. This study aimed to investigate the mechanisms underlying the atheroprotective effect of bixin, a carotenoid obtained from the seeds of the tropical plant *Bixa orellana*, on Cu^2+^-induced LDL oxidation and oxLDL-mediated effects in J774A.1 macrophage cells. Bixin’s effects were compared to those of lycopene, a carotenoid widely studied for its cardiovascular protective effects. LDL was isolated from human plasma, incubated with bixin or lycopene (positive control), and subjected to oxidation with CuSO_4_. Afterward, bixin or lycopene was incubated with J774A.1 macrophage cells and exposed to oxLDL. The levels of ROS, RNS, GSH, nitrite, mitochondrial function, and foam cell formation, as well as the expression of proteins related to the antioxidant and inflammatory status, were evaluated. The effect of bixin in inhibiting in vitro human-isolated LDL oxidation was more potent (5–6-fold) than that of lycopene. Bixin pretreatment reduced the atherogenic signaling triggered by oxLDL in the macrophages, namely the generation of reactive species, disturbance of nitric oxide homeostasis, mitochondrial dysfunction, and foam cell formation. The cytoprotective effects of bixin were accompanied by the upregulation of Nrf2 and the downregulation of the NF-kB pathways. Lycopene showed the same protective effect as bixin, except that it did not prevent mitochondrial dysfunction. The efficient performance of bixin makes it an ideal candidate for further trials as a new nutraceutical compound for the prevention of atherosclerosis.

## 1. Introduction

Atherosclerosis is a leading cause of cardiovascular disease (CVD) [1]. It is a progressive disorder characterized by endothelial dysfunction, proliferation of smooth muscle cells (SMCs) and accumulation of lipids in the arterial wall, leading to macrophage foam cell formation [2]. Oxidative modifications of low-density lipoprotein (LDL) are crucial in the development and progression of atherosclerosis [3]. Vascular oxidative stress enhances the generation of reactive species (RS) by SMCs, endothelial cells, and macrophages, leading to LDL oxidation and its subsequent uptake by macrophages [4]. The uptake oxidized LDL (oxLDL) by macrophages stimulate the production of inflammatory mediators, such as cytokines, proteases, RS, metalloproteinases and other factors, through redox signaling pathways [2].

The balance between the generation of pro-oxidants and the levels of antioxidant defenses in the vessel wall plays a key role in the pathogenesis of atherosclerosis [5]. In this context, lipid-soluble dietary antioxidants have been shown to be incorporated into the LDL structure, reducing its susceptibility to being oxidized [6]. Diets rich in carotenoid-containing fruits and vegetables have been associated with reduced risk of chronic diseases, such as CVD [7]. Among the lipid-soluble pigments, lycopene is one of the major carotenoids found in Western diets [6]. A recent meta-analysis demonstrated a positive correlation between lycopene intake and a reduced incidence of CVD [8], whereas the serum lycopene levels were inversely related to the intimal wall thickness or lesions in the carotid artery, suggesting its promising atheroprotective effect [9]. Lycopene was able to protect LDL from oxidation in vitro, and the intake of lycopene-containing foods increased the resistance of LDL to oxidation in vivo [10]. The beneficial actions of lycopene in relation to health and disease have been attributed to its antioxidant properties [11].

Bixin is a lipophilic carotenoid obtained from the seeds of the tropical annatto plant *Bixa orellana*. Bixin has an excellent safety record [12], is approved by the Food and Drug Administration (FDA) as a natural food colorant (Code of Federal Regulations Title 21, Section 73.30), and has an established systemic bioavailability and pharmacokinetic profile upon oral administration [13,14,15,16]. Moreover, bixin is among the most effective carotenoids to neutralize singlet molecular oxygen (^−1^O_2_), superoxide anion (O_2_^•−^), nitric oxide (^•^NO), and other reactive species [17,18,19], potentially protecting cells and tissues against oxidative damage. Furthermore, bixin’s effects against glutathione (GSH) depletion and lipid peroxidation, as well as its anti-carcinogenic effects [14,20], were associated with its ability to modulate the nuclear factor-E2-related factor 2 (Nrf2)-dependent signaling [14]. This redox-sensitive transcription factor coordinates significant cellular defense mechanisms, such as phase-II detoxification, inflammatory signaling, DNA repair, and the antioxidant response, making it a promising molecular target for therapeutics in various human pathologies [21].

In a previous study, we demonstrated that bixin supplementation attenuated atherosclerotic lesions in hypercholesterolemic rabbits through its anti-inflammatory and antioxidant effect [22]. Bixin also improved the lipid profile in animal models of atherosclerosis [22] and diabetes [23]. Such effects may be related to its agonist action on peroxisome proliferator-activated receptor gamma (PPAR-γ) and peroxisome proliferator-activated receptor alpha (PPAR-α) receptors, which regulate adipogenesis and fatty acid oxidation [24,25].

The present study is carried out to elucidate the general mechanism implicated in the protective effects of bixin in in vitro models related to atherosclerosis. In addition, bixin’s performance will be compared with that of lycopene, which has already emerged as a cardioprotective carotenoid. Other objectives of this study include determining whether bixin is able to protect human-isolated LDL against Cu^2+^-induced oxidation and oxLDL-mediated macrophage cytotoxicity, as well as investigating some molecular pathways implicated in its potential atheroprotective effect.

## 2. Materials and Methods

### 2.1. Reagents

Bixin was sourced from Christian Hansen (Hørsholm, Denmark), while lycopene was obtained from Galena (São Paulo, Brazil). Both bixin and lycopene were dissolved in dimethylsulfoxide (DMSO), serving as a control/vehicle. The concentration of DMSO in the cell culture assays did not exceed 0.05%. All the other chemicals used were of analytical grade and were obtained from standard commercial suppliers.

### 2.2. Human LDL Isolation

This study received approval from the Ethics Committee of the Federal University of Santa Catarina (approval number 943/10, FR 363814). LDL was isolated from fresh human plasma using discontinuous density-gradient ultracentrifugation, following the established protocol [26]. The protein concentration was determined [27] and the LDL was stored at −20 °C for no longer than 2 weeks.

### 2.3. LDL Oxidation Assay

The LDL lipid and protein oxidation were evaluated through conjugated diene formation [28,29] and LDL-tryptophan (TrP) fluorescence [30,31], respectively. The LDL samples (50 µg protein/mL) were pre-incubated for 10 min at 37 °C in a medium containing 10 mM phosphate-buffered saline (PBS), pH 7.4, along with varying concentrations of bixin (0–7.5 µM) or lycopene (0–40 µM).

#### 2.3.1. Measurement of Conjugated Diene Formation

Following pre-incubation with carotenoids, CuSO_4_ (10 µM) was introduced into the reaction medium and the absorbance was continuously measured at 234 nm for 390 min to evaluate the conjugated diene production [28]. The duration of the lag phase (min) was determined as the intercept between the time axis and the tangent of the slope of the absorbance curve in the propagation phase. The oxidation rate (V_max_) was calculated from the slope of the absorbance curve observed during the propagation phase [29].

#### 2.3.2. Measurement of LDL-Tryptophan (TrP) Fluorescence

After pre-incubation with carotenoids, copper sulfate (CuSO_4_; 3.3 µM) was introduced into the reaction medium and the TrP fluorescence was measured at various time points (0–390 min) using a fluorometric microplate reader (excitation at 282 nm and emission at 331 nm). The time (min) required for the half-maximal reduction in the TrP fluorescence (T_max_/2) was calculated and utilized to monitor the Cu^2+^-induced apolipoprotein LDL oxidation [30]. The fluorescence spectra of native LDL exhibit a single band centered at approximately 332 nm, attributed to the TrP residues in apolipoprotein B-100 (ApoB-100), which diminishes upon apolipoprotein oxidation [30,31].

### 2.4. Cell Culture Assays

#### 2.4.1. Cell Culture and oxLDL Preparation

Murine J774A.1 macrophage cells were obtained from the American Type Culture Collection (ATCC, Rockville, MD, USA) and cultured at subconfluence in a 5% CO_2_ humidified atmosphere at 37 °C. The medium utilized for the routine subculture consisted of Dulbecco’s modified Eagle medium (DMEM) supplemented with 2 mM glutamine, 10 mM HEPES, 100 U/mL penicillin, 100 mg/mL streptomycin, and 10% fetal bovine serum (FBS). The cells were subcultured upon reaching confluence (70–80%) and were utilized between the 5th and 15th passages. For the cell exposure to bixin, lycopene and/or oxLDL, the same medium was employed, with the exception that the FBS was limited to 0.5%.

The isolated LDL samples (1 mg of protein/mL) underwent a 16 h incubation with 10 µM CuSO_4_ at 37 °C, leading to the production of oxLDL. Following this, 10 µM ethylenediaminetetraacetic acid (EDTA) was added, and the samples were subsequently dialyzed against PBS (148 mM, pH 7.4) for 24 h at 4 °C. The oxidation status of the LDL was monitored by evaluating the conjugated diene levels, and the resulting oxLDL preparation was stored at −20 °C for a maximum period of 2 weeks.

According to our previous studies, the cells were pretreated with carotenoids for 24 h and then exposed to 100 µg/mL of oxLDL [32].

#### 2.4.2. Cell Viability Assay

The macrophages were cultured in 96-well plates at a density of 1.2 × 10^4^ cells/well and pretreated with either bixin (0.03–30 µM), lycopene (0.001–3 µM), or vehicle for 24 h. The cell viability was determined at 550 nm using the reduction of the 3-(4,5-dimethylthiazol-2-yl)-2,5-diphenyl-tetrazolium bromide (MTT) assay, which evaluates the activity of labile mitochondrial dehydrogenases [33]. The results were expressed as a percentage of the control group (untreated cells).

#### 2.4.3. Measurement of Reactive Species (RS) Production

Intracellular RS production was detected using 2′,7′-dichlorodihydrofluorescein diacetate (DCFH-DA), which permeates the cell, where it is hydrolyzed by cellular esterases and reacts with intracellular RS to form the fluorescent product 2′,7′-dichlorofluorescein (DCF) [34]. To evaluate the antioxidant protective effect of bixin and lycopene against RS production induced by oxLDL, the macrophage cells were plated into 24-well plates at an equal density (2.0 × 10^5^ cells/well) and were pretreated for 24 h with either vehicle, bixin (0.03–0.3 µM), or lycopene (0.001–0.1 µM) at 37 °C. Thereafter, the carotenoid-containing medium was removed and the cells were washed and replaced with Hank’s balanced salt solution (HBSS), consisting of 136 mM sodium chloride (NaCl), 1 mM sodium dihydrogen phosphate (NaH_2_PO_4_), 1.2 mM calcium chloride (CaCl_2_), 1.4 mM magnesium chloride (MgCl_2_), 5.4 mM potassium chloride (KCl), 10 mM HEPES and 9 mM glucose (pH 7.4), containing 10 µM DCFH-DA. The cells were then exposed to oxLDL (100 µg/mL) and the time course of the RS production was immediately evaluated at 37 °C for 60 min with 485 nm excitation and 520 nm emission using a fluorescence plate reader [32]. The results were expressed as the intensity of the fluorescence (arbitrary units) and the oxidation rate was calculated.

#### 2.4.4. Dihydrorhodamine (DHR) Oxidation

DHR oxidation is widely used to measure peroxynitrite (ONOO-) production in vitro as this fluorescent probe reacts with peroxynitrite-derived free radicals but not with O_2_^•−^ or ^•^NO directly [35,36]. The macrophage cells were grown in 24-well culture plates at an equal density (2.0 × 10^5^ cells/well) and pretreated with either vehicle, bixin (0.03–0.3 µM), or lycopene (0.001–0.1 µM) for 24 h. The carotenoid-containing medium was removed and the cells were washed and replaced with Dulbecco’s phosphate-buffered solution (dPBS), consisting of 137 mM NaCl, 8.1 mM Na_2_HPO_4_, 0.9 mM CaCl_2_, 0.5 mM MgCl_2_, 2.7 mM KCl, and 1.45 mM potassium dihydrogen phosphate (KH_2_PO_4_), pH 7.4, supplemented with 5.6 mM glucose, 1 mM L-arginine, and containing 10 µM DHR. The cells were then exposed to oxLDL (100 µg/mL) and the detection of rhodamine 123, an oxidation product of DHR, immediately followed at 37 °C for 60 min at 485 nm excitation and 525 nm and 590 nm emission in a fluorescence plate reader [37]. The results were expressed as the intensity of the fluorescence (arbitrary units) and the DHR oxidation rate was calculated.

#### 2.4.5. High-Resolution Respirometry of Intact Cells

The mitochondrial function was assessed using an Oroboros Oxygraph-O_2_K respirometer (Oroboros Instruments, Innsbruck, Austria), following the protocols outlined by Hort et al. (2014) [38] and the standard guidelines provided by the manufacturer. The macrophage cells were pretreated with either vehicle, bixin (0.03 µM), or lycopene (0.003 µM) for 24 h. Subsequently, they were exposed to oxLDL (100 µg/mL) for another 24 h. After this period, the cells (5.0 × 10^5^ cells/mL) were harvested, centrifuged at 1200× *g* for 3 min, and then resuspended in DMEM without FBS. The O_2_ consumption of the intact cells was measured in the closed (2 mL) respirometer chamber.

The experimental procedure was initiated by measuring the routine respiration, characterized by the respiration in the cell-culture medium devoid of additional substrates or effectors. Following the assessment of the routine respiratory flux, inhibition of adenosine-5′-triphosphate (ATP) synthase was carried out using oligomycin (1 µg/mL) to estimate the oxygen consumption coupled to ATP synthesis. Next, 100 nM carbonyl cyanide-p-trifluoromethoxyphenylhydrazone (FCCP, a mitochondrial uncoupler) was added to determine the maximal oxygen consumption that the cells can sustain. Upon FCCP titration, the electron transport chain of oxidative phosphorylation was altered (blocked) using rotenone (0.5 µg/mL) and antimycin A (1 µg/mL), with the resulting oxygen flow rate denoted as extra-mitochondrial oxygen consumption. Data acquisition and analysis were conducted using DatLab 6 software (Oroboros Instruments, Innsbruck, Austria). The respiratory control ratio (RCR) was calculated as the ratio between the maximal oxygen consumption (FCCP) and leak respiration (oxygen consumption after adding oligomycin). The ATP-linked O_2_ consumption, i.e., coupled respiration, was calculated as the routine respiratory flux minus leak respiration. The reserve capacity was calculated as the maximal oxygen consumption rate minus the routine respiratory rate. The cellular respiration was quantified in terms of the oxygen flux (JO_2_) derived from the rate of change of the O_2_ concentration in the chambers. The ATP-linked O_2_ consumption and reserve capacity were expressed as a percentage of the control (vehicle-treated cells).

#### 2.4.6. Cellular Reduced Glutathione (GSH) Levels

The macrophage cells (2.5 × 10^5^ cells/well) were seeded in 24-well plates and pre-incubated with either vehicle, bixin (0.03 µM), or lycopene (0.003 µM) for 24 h. The cells were then stimulated with oxLDL (100 µg/mL) for an additional 24 h. The GSH intracellular levels were determined using the fluorescent probe *o*-phthalaldehyde, with excitation at 350nm and emission at 420 nm [39]. The quantity of GSH was calculated utilizing a standard curve of reduced glutathione and adjusted with the cellular protein content measured via the Lowry assay [27].

#### 2.4.7. Nitrite Assay

The macrophage cells were cultured in 24-well plates at a density of 2.5 × 10^5^ cells/well. The cells were then pre-incubated with either vehicle, bixin (0.03 µM), or lycopene (0.003 µM) for 24 h. Following this pre-incubation, oxLDL (100 µg protein/mL) was added to the cells, and they were further incubated for an additional 24 h. The supernatants were collected to quantify the nitrite levels at 540 nm after the Griess reaction [40]. The quantity of nitrite (µM) was determined from a standard curve using freshly prepared sodium nitrite in culture medium. The nitrite amount was used as an estimate of the ^•^NO level as it is generated during the oxidative metabolism of ^•^NO.

#### 2.4.8. Inducible Nitric Oxide Synthase (iNOS) Protein Expression

For the Western blotting analysis for the evaluation of the expression of iNOS, macrophages were seeded in 6-well plates at a density of 5.0 × 10^5^ cells/well. Subsequently, they were pre-incubated with either vehicle, or bixin (0.03 µM), or lycopene (0.003 µM) for 24 h, followed by stimulation with oxLDL (100 mg protein/mL) for an additional 24 h. The cells were then washed twice with 1 × PBS and harvested in 200 µL of ice-cold lysis buffer (50 mM Tris-HCl pH 7.5, 1% Triton X-100, 100 mM NaCl, 5 mM EDTA pH 8.0, 40 mM β-glycerophosphate, 50 mM sodium fluoride (NaF), 200 µM sodium orthovanadate (Na_3_VO_4_), 5% glycerol, and protease inhibitors), followed by sonication for 45 s. The samples were centrifuged at 13,000× *g* at 4 °C for 45 min, and the supernatant was collected for the protein measurement [27]. Fifty micrograms of protein extract were loaded onto 7.5% sodium dodecyl sulfate (SDS) polyacrylamide gel electrophoresis (PAGE) and transferred to nitrocellulose membranes. After blocking the membrane with 5% low-fat dried milk in TBS for 1 h, they were incubated overnight at 4 °C with rabbit polyclonal anti-iNOS antibody (1:1000; sc-8310, Santa Cruz Biotechnology, Dallas, TX, USA) or mouse polyclonal anti-β-actin (1:2500; sc-47778, Santa Cruz Biotechnology, Dallas, TX, USA). Following this, they were probed with horseradish peroxidase (HRP)-conjugated secondary antibodies and visualized using the ECL system (GE Healthcare, Chicago, IL, USA) [32]. The membranes were scanned using ChemiDoc MP (Bio-Rad, Hercules, CA, USA), and the band intensity was quantified with Quantity One software (version 6.0.0, Bio-Rad, Hercules, CA, USA) and normalized with respect to the β-actin bands.

#### 2.4.9. Nrf2 and Nuclear Factor Kappa B (NF-κB) Protein Expression

Macrophages were seeded in 6-well plates at a density of 5.0 × 10^5^ cells/well and pre-incubated with either vehicle, or bixin (0.03 µM), or lycopene (0.003 µM) for 24 h. Following this, they were stimulated with oxLDL (100 mg protein/mL) for an additional 24 h. The cells were then washed twice with 1 × PBS and harvested in 200 µL of cold buffer A, consisting of 10 mM HEPES (pH 7.9), 10 mM KCl, 2 mM MgCl_2_, 1 mM EDTA, 2 mM Na_3_VO_4_, 1% Triton X-100 and protease inhibitor, and the lysate were left on ice for 15 min. Afterward, the cell lysates were centrifuged at 15,000× *g* at 4 °C for 30 min, and the supernatants were collected (cytoplasmic fraction). The pellets were then homogenized in cold buffer B, consisting of 20 mM HEPES (pH 7.9), 50 mM KCl, 2 mM MgCl_2_, 420 mM NaCl, 1 mM EDTA, 2 mM Na_3_VO_4_, 1% Triton X-100, 25% glycerol and, protease inhibitor. The samples were sonicated, followed by centrifugation at 15,000× *g* at 4 °C for 30 min, and the resulting supernatant was collected as the nuclear fraction for the protein measurement [27]. Electrophoresis was conducted with 12% SDS/PAGE and Western blotting was performed as in Section 2.4.8. The membranes were incubated with polyclonal rabbit anti-Nrf2 antibody (1:250; sc-722, Santa Cruz Biotechnology, Dallas, TX, USA), mouse monoclonal anti-NF-κB antibody (1:100; sc-166588, Santa Cruz Biotechnology, Dallas, TX, USA), mouse polyclonal anti-β-actin (1:2500; sc-47778, Santa Cruz Biotechnology, Dallas, TX, USA) or mouse monoclonal anti-PCNA (1:200; sc-56, Santa Cruz Biotechnology, Dallas, TX, USA). The band intensity was normalized with respect to the β-actin (cytoplasmic fraction) or proliferative nuclear cell antigen (PCNA; nuclear fraction) bands.

#### 2.4.10. Foam Cell Formation Assay

Macrophages (3.0 × 10^5^ cells/well) were plated on a coverslip in 12-well plates and pretreated with either vehicle, bixin (0.03 µM), or lycopene (0.003 µM) for 24 h. Following pretreatment, the cells were stimulated with oxLDL (100 µg/mL) for an additional 3 h. After incubation with oxLDL, the cells were fixed with 4% paraformaldehyde and stained with 0.3% Oil Red O for 10 min [41]. Hematoxylin was used for the counterstaining. Cell images were captured using light microscopy (Olympus, Tokyo, Japan) with an oil immersion objective (1000 × magnification). Ten images were taken from each group, and the total pixel intensity was quantified using NIH Image J 1.36b imaging software (National Institutes of Health, Bethesda, MD, USA). The lipid content was expressed as the optical density (OD).

### 2.5. Statistical Analysis

The results are presented as the means ± SEM. The GSH, nitrite, respirometry and Western blotting data were analyzed using a two-way analysis of variance (ANOVA), followed by Tukey’s test when appropriate. The remaining data were analyzed using a one-way ANOVA followed by Dunnet’s test (only for MTT data) or Tukey’s test (other data). Linear regression was performed to identify the concentration-dependent effects. Differences were considered significant when *p* < 0.05. All the analyses were performed using the Statistica^®^ 7.0 software system (TIBCO Software Inc, Palo Alto, CA, USA).

## 3. Results

### 3.1. Bixin Is More Potent Than Lycopene in Inhibiting LDL Oxidation

**Figure 1:**
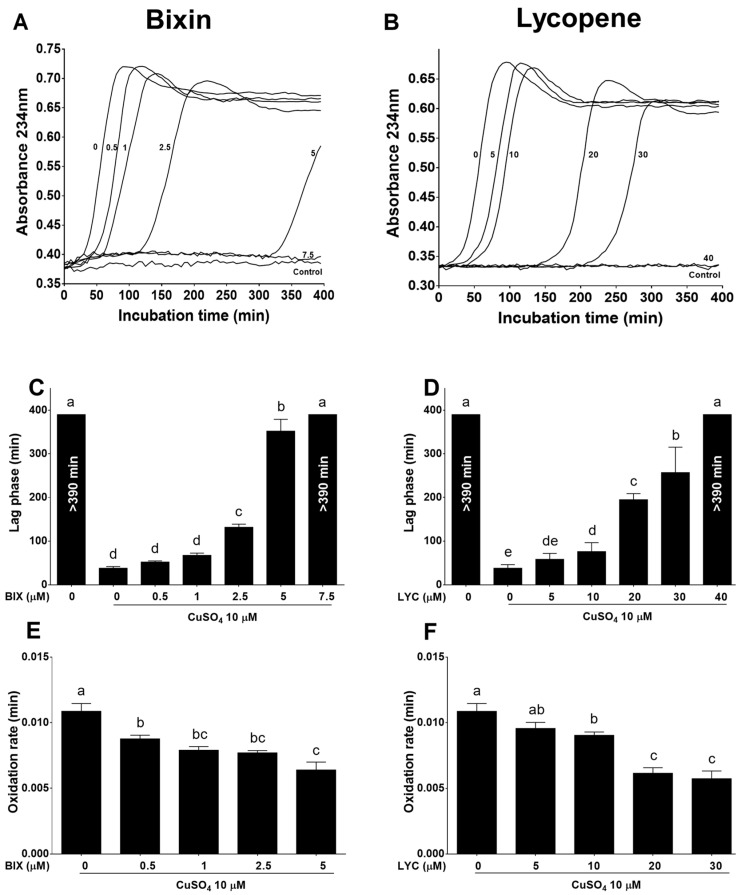
Bixin (**A**,**C**,**E**) is more potent than lycopene (**B**,**D**,**F**) in preventing Cu^2+^-induced lipid peroxidation in isolated human LDL. Representative profile of conjugated dienes formation (**A**,**B**), lag phase (**C**,**D**), and oxidation rate (**E**,**F**) of conjugated dienes formation. In panels (**A**,**B**), the numbers over the lines indicate the carotenoid concentration (µM). Data from panels (**C**–**F**) are the mean ± SEM of three independent experiments. Bars that have no common letters differ significantly (*p* < 0.05). Moreover, >390 min indicates a lag phase higher than the duration of the assay. BIX: bixin; LDL: low-density lipoprotein; LYC: lycopene.

Incubation of native human LDL with Cu^2+^ led to the oxidation of LDL polyunsaturated fatty acids (PUFAs), evidenced by the formation of conjugated diene (Figure 1). The oxidation kinetic profile exhibited an initial lag phase, followed by a propagation phase characterized by a maximal conjugated diene formation rate, and finally a decomposition phase. Both bixin and lycopene inhibited Cu^2+^-induced lipid oxidation of isolated LDL (Figure 1A,B). Bixin in the range of 2.5–7.5 µM (Figure 1C) and lycopene in the range 10–40 µM (Figure 1D) extended the lag phase of Cu^2+^-induced LDL oxidation (*p* < 0.05), and this effect was concentration-dependent for both compounds (bixin: r^2^ = 0.979, *p* < 0.005; lycopene: r^2^ = 0.985, *p* < 0.005; Figure S1A,B, Supplementary Materials). Additionally, bixin 0.5–5 µM (Figure 1E) and lycopene 10–30 µM (Figure 1F) decreased the oxidation rate—V_max_ (*p* < 0.05), and this effect was also concentration-dependent for both compounds (bixin: r^2^ = −0.96, *p* < 0.05; lycopene: r^2^ = −0.96, *p* < 0.05; Figure S1C,D, Supplementary Materials). The presence of 7.5 µM bixin and 40 µM lycopene almost completely abolished Cu^2+^-induced lipid oxidation during an assay time of 390 min (Figure 1A and Figure 1B, respectively).

**Figure 2:**
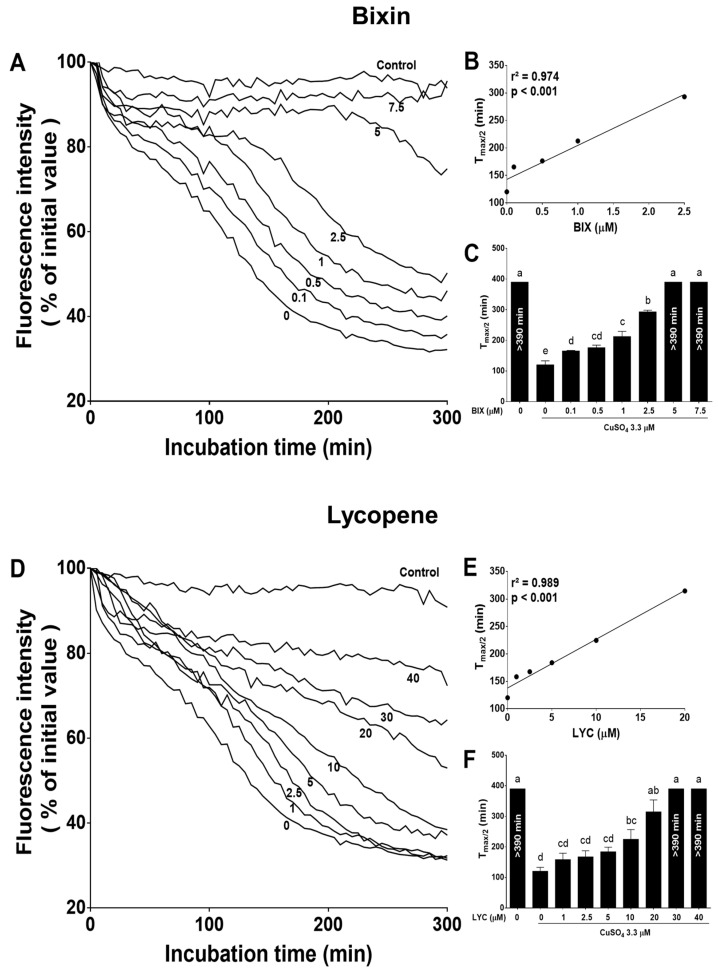
Bixin (**A**–**C**) is more potent than lycopene (**D**–**F**) in preventing Cu^2+^-induced tryptophan oxidation in isolated human LDL. Representative profile of the loss of tryptophan fluorescence (**A**,**D**), average values (**C**,**F**) and linear regression (**B**,**E**) for the time required for the half-maximal reduction in tryptophan fluorescence (T_max_/2). In panels (**A**,**D**), the data are the percentage of emission intensity before Cu^2+^ addition and the numbers under the lines indicate the carotenoid concentration (µM). The data from panels (**B**,**C**,**E**,**F**) are the mean ± SEM of three independent experiments. Bars that have no common letters differ significantly (*p* < 0.05). Moreover, >390 min indicates a lag phase higher than the duration of the assay. BIX: bixin; LYC: lycopene.

In addition to lipid oxidation, exposure of LDL to Cu^2+^ also induced protein oxidation, evident as a decrease in the tryptophan fluorescence (Figure 2). Both bixin and lycopene inhibited this loss of tryptophan fluorescence (Figure 2A,D). The time required to decrease 50% of the tryptophan fluorescence (T_max_/2) was increased in the presence of 0.1–2.5 µM bixin (Figure 2C) and 10–20 µM lycopene (Figure 2F), and it was completely inhibited in the presence of 5–7.5 µM bixin (Figure 2C) and 30–40 µM lycopene (Figure 2F) within 390 min (*p* < 0.05). This effect was concentration-dependent for both carotenoids (bixin: r^2^ = 0.974, *p* < 0.001; lycopene: r^2^ = 0.989, *p* < 0.001; Figure 2B and Figure 2E, respectively). It is noteworthy that bixin was five times more potent than lycopene in completely inhibiting LDL lipid oxidation and six times more potent than lycopene in completely inhibiting LDL protein oxidation.

### 3.2. Effect of Bixin and Lycopene on Macrophage Cell Viability

To evaluate the beneficial effects of bixin or lycopene on oxLDL-mediated toxicity in macrophage cells, we first determined the non-toxic concentration of bixin or lycopene. After 24 h of exposure of the macrophage cells to bixin (0.03–30 µM) or lycopene (0.001–3 µM), we established a concentration-response curve of cell viability. Both carotenoids caused a concentration-dependent decline in the metabolic ability to reduce MTT, with significant effects being detected at concentrations equal to or higher than 1 µM bixin and 0.03 µM lycopene (Figure S2A,B, Supplementary Materials), respectively (*p* < 0.05). Based on these results, the subsequent cell culture studies were conducted at maximal concentrations of 0.3 µM for bixin and 0.01 µM for lycopene, which did not induce the loss of cell viability within 24 h.

### 3.3. Bixin and Lycopene Prevent the Generation of Oxidative Species Triggered by oxLDL in Macrophage Cells

**Figure 3:**
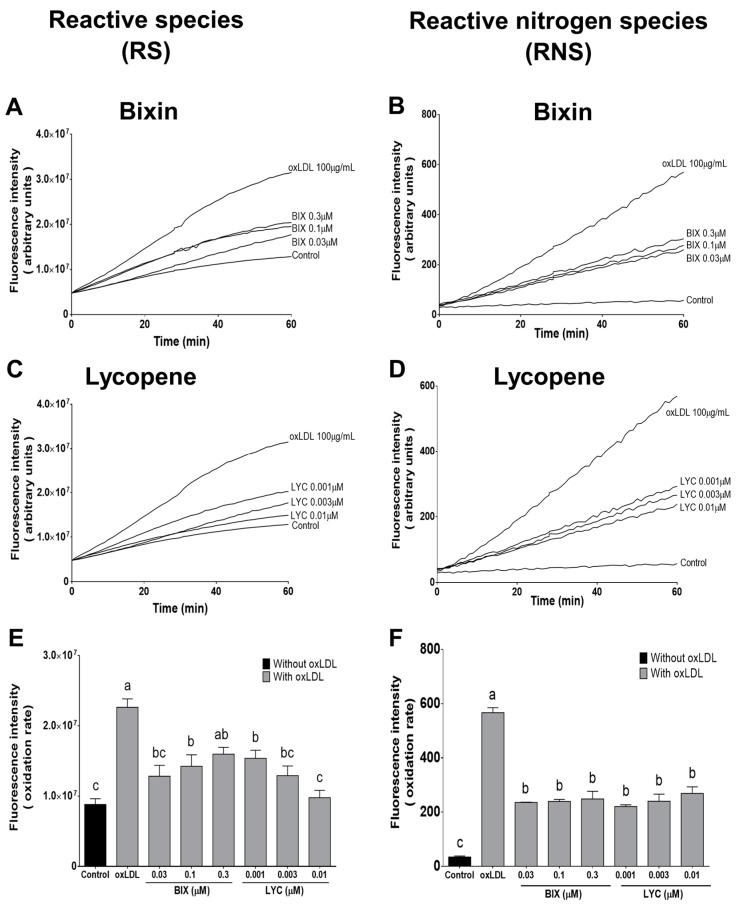
Bixin and lycopene decrease the intracellular production of reactive species (RS) and reactive nitrogen species (RNS). Representative profile of the RS (**A**,**C**) and RNS (**B**,**D**) generation monitored for 1 h in macrophages cells pretreated with bixin or lycopene for 24 h and after exposure to oxLDL. Amount of intracellular production of RS and RNS (**E**,**F**). Each bar represents the mean ± SEM of three independent experiments. Bars that have no common letter are significantly different (*p* < 0.05). BIX: bixin; LYC: lycopene; oxLDL: oxidized low-density lipoprotein.

Besides being involved in the formation of oxLDL, intracellular RS generation plays a key role in the cytotoxic effects of oxLDL [4]. The intracellular fluorescent probes DCF-DA and DHR were used to assess the time course of RS and RNS production in macrophage cells exposed to oxLDL. Exposure of the macrophage cells to oxLDL (100 µg/mL) induced exponential RS generation up to 60 min (Figure 3A,C), and pretreatment of the cells with bixin (0.03–0.1 µM) or lycopene (0.001–0.01 µM) for 24 h efficiently reduced the intracellular RS production (*p* < 0.05; Figure 3E).

RNS, such as ONOO^−^, play a significant role in the nitroxidation of biomolecules, especially in relation to mitochondria [42,43]. Exposure of macrophage cells to oxLDL (100 µg/mL) also triggered the production of ONOO^−^-derived radicals herein assessed by DHR oxidation (Figure 3B,D). Rhodamine formation after oxLDL-exposure was prevented by pretreatment of the cells with all the bixin and lycopene concentrations for 24 h (*p* < 0.05, Figure 3F), demonstrating that these compounds can prevent DHR oxidation by secondary radicals derived from ONOO^−^ at very low concentrations.

Based on these results, pretreatment for 24 h with 0.03 µM bixin or 0.003 µM lycopene was chosen to investigate their protective mechanisms against the cytotoxic effects of oxLDL in macrophages, as described below.

### 3.4. Effect of Bixin in oxLDL-Induced Mitochondrial Dysfunction

**Figure 4:**
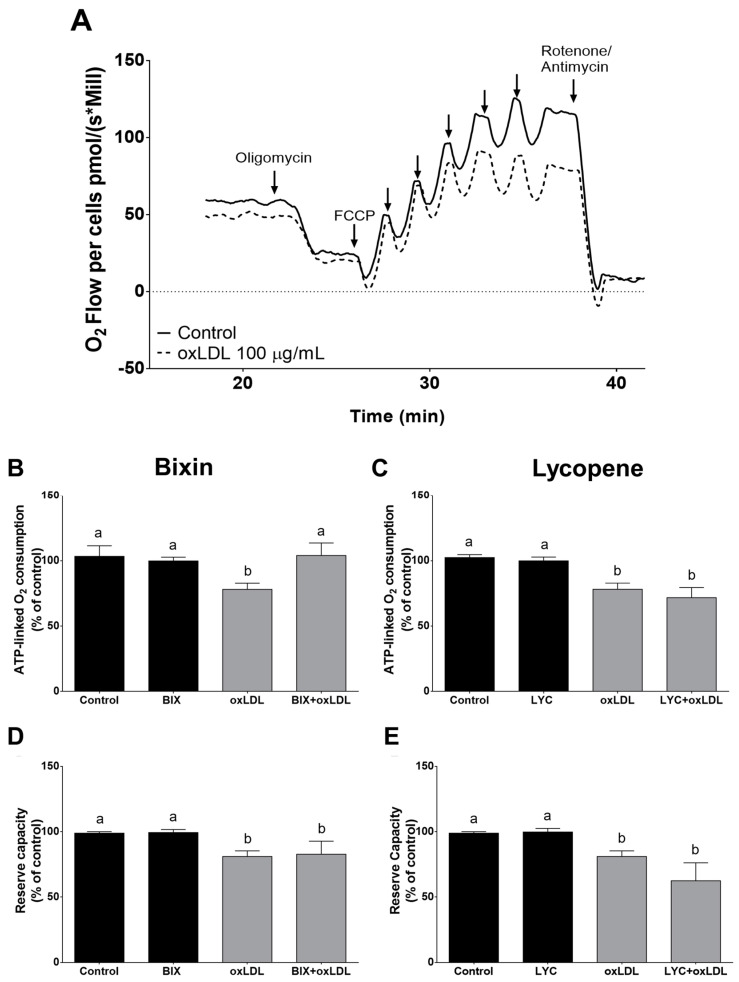
Bixin, but not lycopene, prevents the macrophage mitochondrial dysfunction induced by oxLDL (**A**–**E**). Representative record of the oxygen flow in macrophage cells exposed or not to oxLDL for 24 h (**A**). ATP-linked O_2_ consumption (i.e., coupled respiration = routine respiration − leak respiration) (**B**,**C**). Reserve capacity (maximal respiratory consumption rate − routine respiratory rate) (**D**,**E**). The arrows in panel (**A**) show the steps in the titration regime with FCCP. The respiratory states are indicated as: routine, routine state in cell culture medium; oligomycin (1 mg/mL), inhibition of ATP synthase; FCCP (100 nM per injection), maximal stimulation by uncoupling of oxidative phosphorylation in subsequent titrations of FCCP; and rotenone (0.5 µg/mL)/antimycin A (1 µg/mL), stopping/blocking the electron transport chain of oxidative phosphorylation. Each bar represents the mean ± SEM of three independent experiments. Bars that have no common letters are significantly different (*p* < 0.05). BIX: bixin; LYC: lycopene; FCCP: carbonyl cyanide-p-trifluoromethoxyphenylhydrazone; oxLDL: oxidized low-density lipoprotein.

OxLDL has been shown to negatively impact various cellular functions, including mitochondrial respiratory activity and RS generation [44]. High-resolution respirometry, conducted using Oroboros, enabled the detection of subtle changes in the O_2_ flux within the electron transport system, whether coupled to ATP synthesis or not. Initially, the O_2_ consumption rates were measured in intact macrophage cells treated or untreated with oxLDL (100 µg/mL) (Figure 4A). Reductions in both the baseline and maximum respiration were observed in macrophages exposed to oxLDL for 24 h (Figure 4A). Oligomycin-inhibited respiration represents O_2_ consumption uncoupled to ATP synthesis, which is termed leak respiration. By inhibiting ATP synthase with oligomycin, we calculated the ATP-coupled respiration (routine respiratory flux minus leak respiration). The ATP-coupled respiration was significantly reduced by oxLDL (Figure 4B,C), suggesting potential inefficiency in ATP synthesis. Pretreatment of the cells with bixin prevented disruption of the ATP-coupled respiration, whereas lycopene showed no effect (Figure 4B,C). When the proton ionophore FCCP was added to the cells, it dissipated the proton gradient, maintaining the mitochondrial oxygen flow at a maximum rate; the difference between the maximum respiratory capacity and the routine respiration represents the potential cellular reserve capacity. OxLDL exposure led to a significant reduction in the reserve capacity of the macrophages (Figure 4D,E, *p* < 0.05), while pretreatment with bixin or lycopene did not prevent this effect. Neither bixin nor lycopene alone altered any mitochondrial parameter evaluated in this study. The respiratory control ratio (RCR), reflecting the efficiency of mitochondrial respiration coupled or not to ATP synthesis, remained unchanged after carotenoids or oxLDL exposure.

### 3.5. Bixin Increases GSH Content and Activates Nrf2 in Macrophage Cells

**Figure 5:**
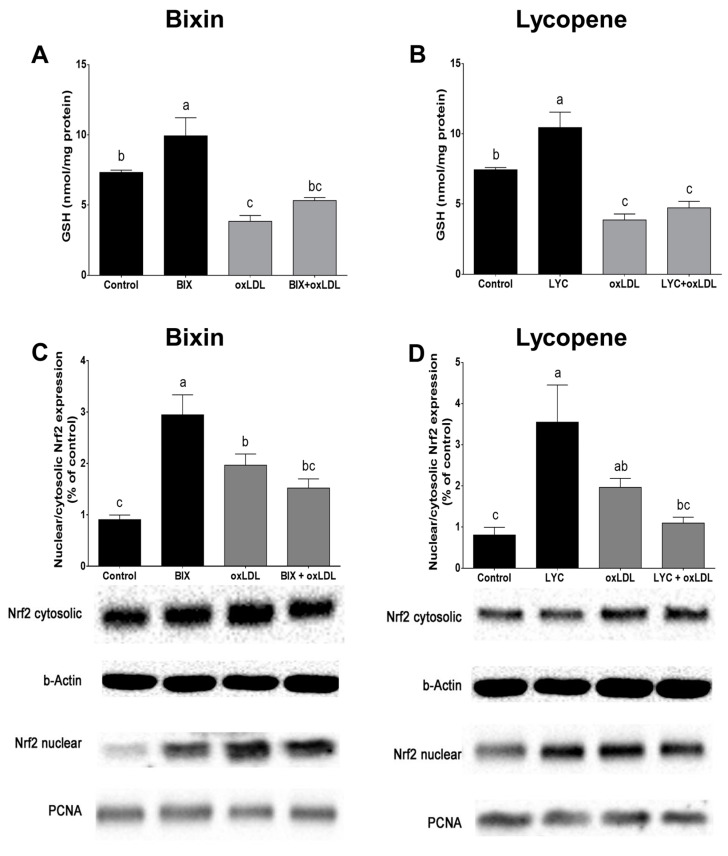
Effect of bixin (**A**) and lycopene (**B**) on the cellular reduced glutathione (GSH) content and immunocontent of nuclear factor-E2-related factor 2 (Nrf2; (**C**,**D**)) in macrophage cells exposed to oxLDL. Quantification of the bands for the nuclear/cytosolic Nrf2 ratio protein expression and representative Western blotting analysis of the cytoplasmic and nuclear expression of Nrf2 ((**C**), bixin; (**D**), lycopene). β-actin was used to normalize the data from the cytoplasmic fraction and proliferative nuclear cell antigen (PCNA) for the nuclear fraction. Data are the mean ± SEM of at least three independent experiments. Bars that have no common letters are significantly different (*p* < 0.05). BIX: bixin; LYC: lycopene; oxLDL: oxidized low-density lipoprotein.

To elucidate the mechanisms underlying the protective effect of bixin and lycopene, we investigated carotenoids’ effect on the macrophage GSH content and the activation of the Nrf2 pathway. Pretreatment with bixin (0.03 µM) and lycopene (0.003 µM) for 24 h increased the GSH content in the macrophage cells (*p* < 0.05, Figure 5A,B). Exposure to oxLDL significantly decreased the intracellular GSH content. Bixin pretreatment partially prevented such an effect, while lycopene had no effect in preventing oxLDL-induced GSH depletion (*p* < 0.05).

The nuclear levels of the Nrf2 factor, a pivotal transcription factor that regulates the expression of antioxidant/phase II detoxification enzymes, exhibited robust augmentation in the macrophage cells following a 24 h of treatment with either bixin or lycopene (Figure 5C,D). Notably, exposure to oxLDL (100 µg/mL) for the same duration also triggered Nrf2 activation in the macrophage cells, evident from the increased nuclear/cytosolic Nrf2 ratio (*p* < 0.05). This effect, however, was partially mitigated by pretreatment with bixin or lycopene (*p* < 0.05; Figure 5C,D).

### 3.6. Bixin and Lycopene Block ^•^NO Production and NF-κB Activation Triggered by oxLDL

**Figure 6:**
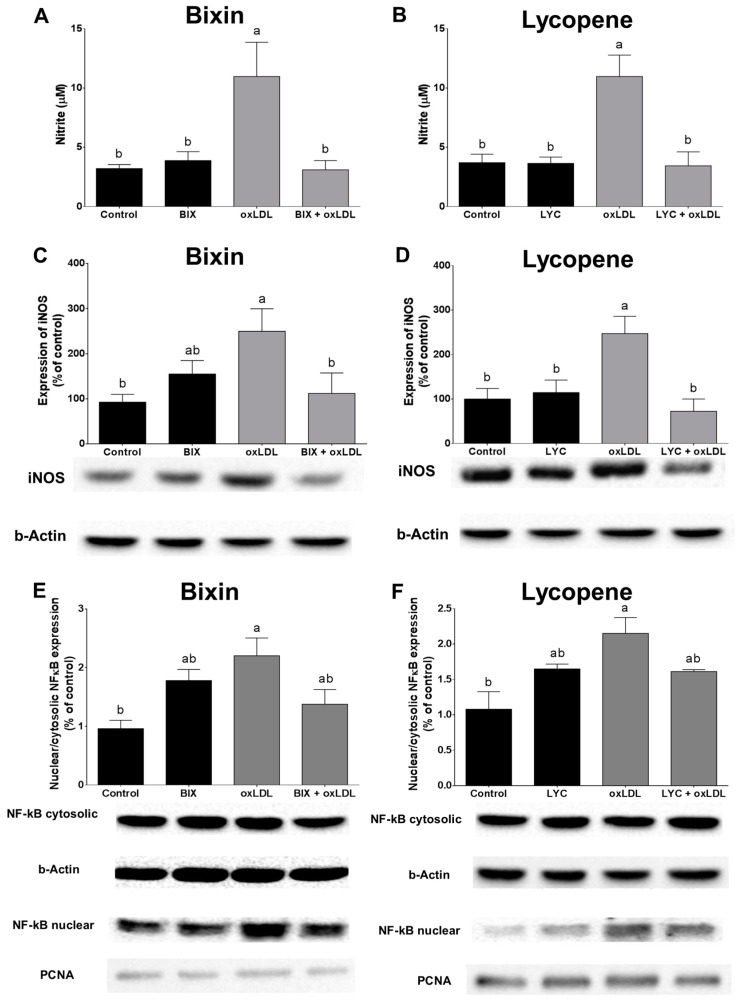
Bixin and lycopene prevent oxLDL-induced nitrite production (**A**,**B**), protein expression of inducible nitric oxide synthase (iNOS), and activation of nuclear factor kappa B (NF-κB) in macrophages cells (**C**–**F**). Band quantification and representative Western blotting analysis of iNOS expression (**C**,**D**) and of NF-κB cytoplasmic and nuclear expression ((**E**), bixin; (**F**), lycopene). β-actin was used to normalize the data for the iNOS and NF-κB cytoplasmic fraction and the proliferative nuclear cell antigen (PCNA) for NF-κB nuclear fraction. Results are the mean ± SEM of three independent experiments. Bars that have no common letters are significantly different (*p* < 0.05). BIX: bixin; LYC: lycopene; oxLDL: oxidized low-density lipoprotein.

The activation of iNOS under inflammatory conditions mediates excessive ^•^NO synthesis [35]. Exposure to oxLDL (100 µg/mL) increased the protein expression of iNOS, with a consequent increase in the ^•^NO levels in the macrophages (*p* < 0.05), and such effects were prevented by pretreatment with bixin or lycopene (*p* < 0.05, Figure 6). The macrophages’ exposure to oxLDL (100 µg/mL) for 24 h also activated NF-κB, as observed by the increased nuclear/cytosolic NF-κB ratio protein expression (*p* < 0.05), and pretreatment with bixin or lycopene partially prevented nuclear translocation of the NF-κB protein (Figure 6E,F).

### 3.7. Bixin and Lycopene Reduced oxLDL-Induced Foam Cells Formation

**Figure 7:**
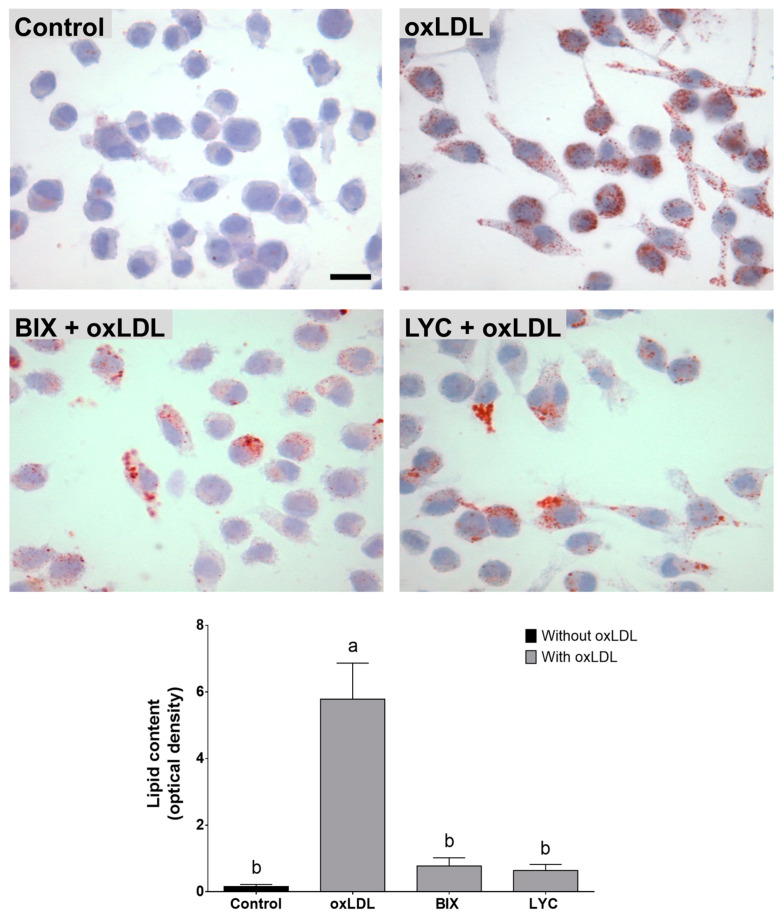
Bixin and lycopene inhibit foam cell formation induced by the exposure of macrophages to oxLDL. Representative images of Oil Red O staining in macrophages (magnification 100×; scale bar 10 µm). In the panel, the quantification results of the intracellular lipid content (optical density) are the mean ± SEM of three independent experiments. Bars with different letters differ significantly (*p* < 0.05). BIX: bixin; LYC: lycopene; oxLDL: oxidized low-density lipoprotein.

When the macrophages were exposed to oxLDL (100 µg/mL), it led to the uptake of this oxidized lipoprotein and the subsequent formation of foam cells, as evidenced by the increased intracellular lipid content (*p* < 0.05). Remarkably, this phenomenon was prevented by pretreatment with bixin (0.03 µM) or lycopene (0.003 µM) (*p* < 0.05; Figure 7).

## 4. Discussion

In this study, we showed the promising effects of the carotenoids bixin and lycopene in preventing several oxidation and inflammatory events triggered by LDL oxidation. Both carotenoids were efficient in preventing LDL oxidation, reflecting their direct antioxidant effect. Moreover, they were able to modulate the intracellular antioxidant and anti-inflammatory pathways in macrophages, rendering the cells more resistant to oxidative cytotoxicity induced by oxLDL. Our data contribute to clarifying the mechanisms involved in the anti-atherogenic properties of these widely consumed carotenoids.

The oxidation process of LDL lipids begins with the extraction of a hydrogen atom from a methylene group of LDL PUFAs, resulting in the formation of a carbon-centered radical that is highly reactive and undergoes molecular rearrangement to achieve a more stable configuration, known as a conjugated diene [45]. Subsequently, these conjugated dienes react with molecular oxygen to generate peroxyl radicals, which then scavenge the hydrogen atom from the adjacent PUFA molecules, thus perpetuating the chain reaction of lipid oxidation [4]. A widely used method for assessing LDL oxidation in vitro involves measuring the formation of conjugated dienes in response to peroxidative modification of the LDL PUFAs mediated by Cu^2+^ ions. This approach, employing isolated human LDL, is based on previous research findings that LDL oxidation in a cell-free system induced by redox-active metal ions like copper and iron closely mimics the physiological and biochemical characteristics in cellular systems. It provides crucial insights into the direct antioxidant activity of potential anti-atherogenic compounds. In the present study, bixin and lycopene exhibited a concentration-dependent inhibition of Cu^2+^-induced lipid peroxidation in isolated human LDL by reducing the rate of oxidation. The lag phase is indicative of the inherent resistance of LDL to oxidation and is widely utilized to assess the potential anti-atherogenic properties of compounds [3,29,46].

In addition to preventing lipid peroxidation of LDL, bixin and lycopene also prevented protein oxidation of LDL, pointing to an additional mechanism that could contribute to the inhibition of atherogenic process. Oxidation of ApoB-100, which can be monitored by the loss of TrP fluorescence, alters its conformation and facilitates the recognition of oxLDL by scavenger receptors, which uptake oxLDL into macrophages, a fundamental step in the pathogenesis of atherosclerosis [3]. The protective effect on LDL oxidation triggered by bixin can be related to its ability to remove peroxyl radicals [47]. The long polyene chain is responsible for the direct free radical scavenging properties of bixin [17,18]. The direct antioxidant effect of bixin against lipid and protein LDL oxidation occurred at concentrations 5- to 6-fold lower than those required for lycopene, showing that bixin was more efficient in directly inhibiting the oxidation of isolated LDL.

The accumulation of oxLDL in the arterial wall and the recruitment of monocytes to the subendothelial space are recognized as the primary early events in the development of atherosclerosis [1,3]. OxLDL induces the production of intracellular RS and RNS, including ^•^NO, O_2_^•−^, H_2_O_2_, and ONOO-, in monocyte-derived macrophages [48]. These RS, originating from various pathways, such as NADPH-oxidase activation, iNOS, and mitochondrial dysfunction [49], can further activate pro-inflammatory pathways and apoptotic processes [50]. In the present study, bixin efficiently attenuated oxLDL-induced intracellular RS/RNS generation in macrophages, aligning with evidence demonstrating bixin’s ability to prevent oxidative stress in aortic tissue [22]. Similarly, lycopene also reduced oxLDL-induced intracellular RS/RNS generation in macrophages, consistent with its known antioxidant properties, which involve reducing oxLDL formation and neutralizing RS [8,10,15].

Natural and synthetic molecules, such as polyphenols, carotenoids, and isothiocyanates, can collaborate to enhance cellular adaptation to oxidative stress by increasing the capacity to neutralize RS and prevent oxidative damage [51]. This class of antioxidants, termed indirect antioxidants, operate by inducing the cytoprotective genes involved in endogenous antioxidants synthesis and regeneration, as well as by influencing xenobiotic metabolism [5,51]. The reduction in the oxLDL-induced RS/RNS production and foam cell formation by bixin and lycopene may stem from their intracellular indirect effect, i.e., their ability to activate the transcription factor Nrf2. This activation, as observed in this study, leads to increased GSH content. Nrf2 binds to antioxidant responsive elements (AREs), facilitating the transcription of genes involved in GSH synthesis, detoxification enzymes, and other pathways crucial for mitigating oxidative stress [21]. This study reports for the first time the ability of bixin to activate the translocation Nrf2 to the nucleus in macrophage cells. This finding suggests that the Nrf2 translocation induced by bixin triggers the intracellular increase in the GSH content, effectively contributing to an improvement in the redox environment and making macrophages more resistant to oxidative insults. In skin cells, bixin has already promoted an effective Nrf2 nuclear translocation through interaction with the critical cysteine (Cys)-151 sensor residue in Keap-1, a protein that anchors Nrf2 in the cytoplasm [14]. The nuclear translocation of Nrf2 induced by bixin resulted in increased gene expression of gamma-glutamylcysteine synthetase (GGCS), the enzyme responsible for GSH synthesis [14]. This induction of GGCS could explain the observed increase in the intracellular GSH content and supports the notion that bixin acts as an indirect antioxidant, enhancing the cellular environment.

It has been reported that overproduction of ROS and RNS in the vascular wall occurs very early in the atherogenic process, potentially leading to mitochondrial dysfunction in two of the most critical cells involved: endothelial and macrophages cells [32,38,42,52]. Mitochondrial dysfunction induced by ROS and RNS is characterized by the inhibition of mitochondrial respiration and a significant decrease in the cellular ATP levels [44]. Throughout the atherogenic process, the excessive RS produced by oxLDL can oxidize mitochondrial proteins, resulting in mitochondrial dysfunction, bioenergetic impairment, and increased production of additional RS such as O_2_^•−^, which rapidly reacts with ^•^NO to form ONOO^−^ [53]. This process can compromise cell survival by either opening the mitochondrial permeability transition pore or activating the mitochondrial apoptosis pathways [53]. In human macrophages, the oxLDL-induced overproduction of RS impairs mitochondrial function and induces apoptosis [52], a phenomenon closely associated with the formation of foam cells in the early stages of atherogenic process.

A high-resolution real-time respirometry assay in intact cells allows detailed investigation of mitochondrial bioenergetics [44]. Here, exposure to oxLDL reduced macrophages’ O_2_ consumption coupled to ATP synthesis and the respiratory reserve capacity, significantly impacting the cellular bioenergetics. Reduced ATP-linked O_2_ consumption indicates either a low ATP demand, lack of substrate availability, or severe damage to the electron transport chain, impeding the electron flow and resulting in lower O_2_ consumption [44]. The reduced mitochondrial respiratory reserve capacity of macrophages exposed to oxLDL indicates impaired ability to meet the increased energy demands required to counteract oxidative stress, leading to apoptosis through mitochondrial signaling [52]. The decrease in the mitochondrial reserve capacity is a strong indicator of mitochondrial dysfunction [54]. Moreover, the observed increase in leak respiration can be partly attributed to intramitochondrial RS generation, which amplifies the mitochondrial dysfunction when macrophages are incubated with oxLDL. Bixin, but not lycopene, prevented the impaired O_2_ coupled to ATP synthesis caused by oxLDL. In this sense, bixin pretreatment enhances the macrophages’ ability to deal with the increased bioenergetics demands in an oxidative environment.

Bixin’s ability to modulate the antioxidant response and improve the redox balance through the activation of the Nrf2 pathway may protect mitochondrial proteins and other biomolecules from oxidative damage. Although lycopene also activates the nuclear Nrf2 translocation, it was not able to prevent the decrease in the GSH levels. Adequate levels of GSH allow cells to neutralize oxidants and, as a consequence, prevent mitochondrial dysfunction and cell death later [38,42,54]. Thus, lycopene has failed to prevent mitochondrial dysfunction induced by oxLDL in macrophage cells. These findings highlight that bixin could induce more intense and relevant modulations in preventing the atherogenic process compared to lycopene.

^•^NO is known to counteract the atherogenic process by mediating vasorelaxation, reducing vascular smooth muscle cell proliferation, and decreasing platelet aggregation [43]. However, excess ^•^NO can react with O_2_^•−^ to form ONOO^−^, a biologically relevant oxidizing and nitrating agent [35,53]. Studies have demonstrated that oxLDL disrupts ^•^NO homeostasis in macrophages [32] and endothelial cells [37,38,42]. Both bixin and lycopene prevented the increase in iNOS protein expression and the ^•^NO and ONOO^−^ levels caused by the macrophages’ exposure to oxLDL. Other carotenoids, such as β-carotene and astaxanthin, have also been shown to inhibit ^•^NO production and decrease iNOS expression in macrophages stimulated with lipopolysaccharide (LPS) [55,56]. This effect likely occurs in vivo, as Roehrs and colleagues (2014) showed that bixin reduced the ^•^NO serum levels in diabetic rats, correlating with a decrease in protein oxidation mediated by ONOO^−^ [23].

NF-κB is a redox-sensitive transcription factor that triggers the expression of proinflammatory mediators such as tumor necrosis factor alpha (TNF-α), interleukin 6 (IL-6), iNOS, and CD36 receptor [49]. The interaction between NF-κB and the inhibitory protein of nuclear factor kappa B (IκB) is regulated by redox-sensitive protein kinases containing several cysteine residues [57]. Data from macrophages exposed to minimally oxidized LDL (mmLDL) and LPS revealed that the cooperative engagement of NF-κB results in the additive/synergistic increased transcription of inflammatory cytokines within atherosclerotic lesions [58]. Here, we showed that the exposure of macrophages to oxLDL increased the RS/RNS levels, consequently activating NF-κB and promoting its translocation to the nucleus. However, both bixin and lycopene blocked this nuclear translocation of NF-κB. Furthermore, bixin and lycopene also prevented the oxLDL-induced increase in the iNOS expression and ^•^NO levels, both of which are inflammatory mediators upregulated by NF-κB activation. Additionally, bixin treatment prevented the increase in TNF-α and IL-6, other inflammatory mediators triggered by the activation of NF-κB, in hypercholesterolemic rabbits [22]. Similarly, other carotenoids have been shown to inhibit the production of inflammatory mediators by blocking the nuclear translocation of NF-κB, likely through their inhibitory effect on IκB kinase (IKK) activity and IκB alpha (IκBα) degradation/phosphorylation, attributed to their antioxidant activity [55,56]. We propose that this anti-inflammatory effect of bixin is secondary to the activation of the Nrf2 pathway, as Nrf2 upregulation has been demonstrated to attenuate NF-κB-controlled signaling by increasing the cell redox balance and decreasing RS generation [59].

Macrophage uptake of oxLDL occurs through various cell surface receptors, particularly CD36. This uptake leads to cytotoxicity, stimulation of cholesterol accumulation, and ultimately, the formation of foam cells, which is the hallmark of early atherosclerotic lesions [60]. The expression of the CD36 receptor is not downregulated by the intracellular cholesterol levels and is increased in atherosclerotic lesions due to autoregulation by components of the oxLDL particles, especially lipoperoxides [2,50]. Additionally, it is well established that the RS-induced nuclear translocation of NF-κB upregulates CD36 receptor expression [61]. In this study, we demonstrated that bixin efficiently decreased the oxLDL uptake and subsequent foam cell formation. We propose that this effect is related to bixin’s ability to decrease LDL oxidation and NF-κB activation, which in turn reduces the expression of CD36, resulting in less uptake of oxLDL by macrophages. Decreased foam cell formation is expected to prevent or delay the atherogenic process. Consistently, we have demonstrated that bixin reduces the extent of atherosclerotic plaques in hypercholesterolemic rabbits [22].

Recent studies have shown an inverse relationship between the intake of tomatoes and/or lycopene and the incidence of atherosclerosis and CVD [8,10]. Because lycopene is an efficient antioxidant [11,18], it has been proposed that this property may be responsible for its cardiovascular beneficial effects. Our results show that bixin was more potent than lycopene in increasing LDL resistance to oxidation and both carotenoids inhibited the intracellular RS and RNS production triggered by oxLDL in macrophage cells.

We revealed novel molecular targets for the anti-atherogenic effects of bixin and lycopene. Both compounds prevented foam cell formation and modulated the macrophage antioxidant response by increasing Nrf2 nuclear expression, although only bixin increased the GSH levels. Additionally, these carotenoids reduced the inflammatory response by altering the nuclear expression of NF-κB, iNOS expression and ^•^NO levels. It is worth noting, however, that only bixin prevented mitochondrial dysfunction, and this result suggests that bixin’s ability to protect the mitochondria is dependent on the GSH levels. Importantly, this study represents the first investigation into the effects of carotenoids on mitochondrial dysfunction in macrophages exposed to oxLDL. The modulation of antioxidant and anti-inflammatory events contributed to the reduction of the formation of foam cells in vitro.

## 5. Conclusions

In summary, the effects observed with bixin and lycopene are likely attributed to their direct scavenger capabilities and/or their ability to activate antioxidant-related genes, thereby enhancing the cellular redox response. Bixin exhibited greater potency than lycopene in directly inhibiting lipid and protein oxidation of human LDL. Furthermore, both carotenoids demonstrated the ability to mitigate oxLDL-induced cytotoxicity, macrophage uptake of oxLDL, and foam cell formation. We propose that the cytoprotective effects of both carotenoids stem from their capacity to trigger Nrf2 signaling consequently inhibiting NF-κB activation and inflammation. These findings shed light on the direct antioxidant properties of bixin and lycopene on LDL and the mechanisms involved in protecting macrophages from oxLDL toxicity, thus offering promising avenues for the development of new therapeutic or nutritional strategies for the prevention and treatment of atherosclerosis.

## Data Availability

The original contributions presented in the study are included in the article/supplementary material, further inquiries can be directed to the corresponding author.

## Supplementary Materials

The following supporting information can be downloaded at https://www.mdpi.com/article/10.3390/foods13132002/s1, Figure S1: Linear regression of the lag phase and the Vmax was used in order to verify the concentration-dependent effects of bixin (A and C) and lycopene (B and D); Figure S2: Effect of bixin (A) and lycopene (B) on the cell viability of macrophages determined by MTT assay.
