# Supplementary material for: Bixin, a New Atheroprotective Carotenoid Candidate, Prevents oxLDL-Induced Cytotoxicity and Mitochondrial Dysfunction in Macrophages: Involvement of the Nrf2 and NF-κB Pathways
Source: Foods. 2024 Jun 25;13(13):2002. doi: 10.3390/foods13132002 (PMC11241531; doi:10.3390/foods13132002)
Supplement: Supplementary file 1 [file foods-13-02002-s001.zip › foods-3054183-supplementary.pdf]

# SUPPLEMENTARY MATERIALS

Bixin, the new atheroprotective carotenoid candidate, prevents oxLDL-induced cytotoxicity and mitochondrial dysfunction in macrophages: Involvement of Nrf2 and NF-κB pathways

Sabrina Somacal[1,2], Luana Caroline Schüler da Silva[3], Jade de Oliveira[3,4], Andreza Fabro de Bem[3,5*], Tatiana Emanuelli[1,2]

[1]Graduate Program on Pharmacology, Center of Health Sciences, Federal University of Santa Maria, 97105-9003, Santa Maria, RS, Brazil;
[2]Department of Food Technology and Science, Center of Rural Sciences, Federal University of Santa Maria, 97105-900, Santa Maria, RS, Brazil;
[3]Department of Biochemistry, Federal University of Santa Catarina, 88040-900, Florianopolis, SC, Brazil;
[4]Department of Biochemistry, Federal University of Rio Grande do Sul, 90035-000, Porto Alegre, RS, Brazil;
[5]Department of Physiological Sciences, Institute of Biological Science, University of Brasília, 70910-900, Brasília, DF, Brazil

*Corresponding author: Department of Physiological Sciences, Institute of Biological Science, University of Brasília, 70910-900, Brasília, DF, Brazil. Telephone number: +55 61 3107-2902; E-mail: debemandreza@gmail.com (Andreza Fabro de Bem).

**Figure S1 – Linear regression of lag phase and Vmax was used in order to verify the concentration-dependent effects of bixin (A and C) and lycopene (B and D).**

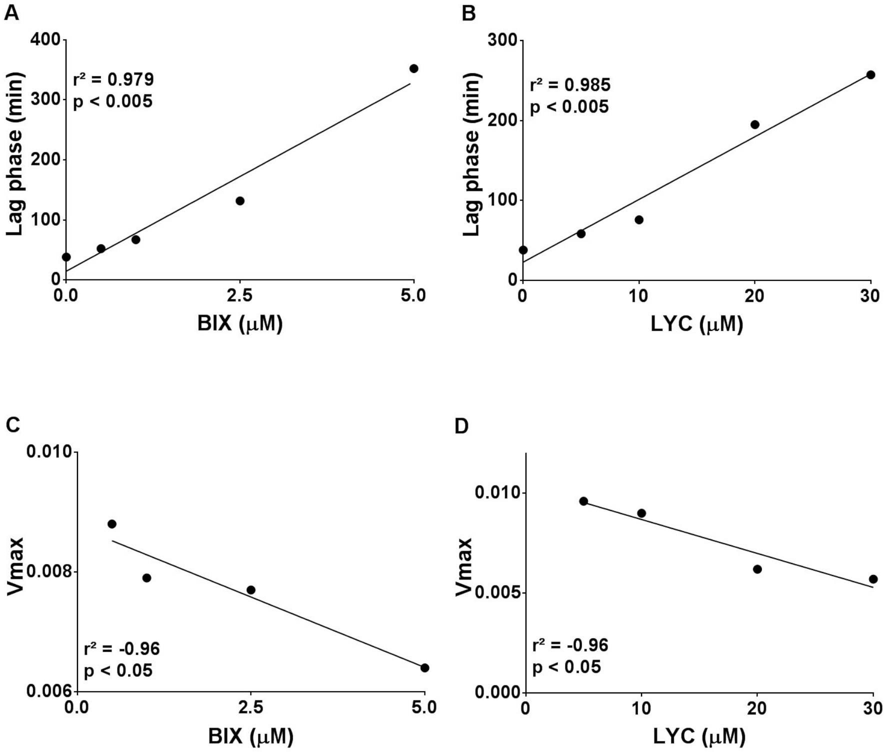


BIX: bixin; LYC: lycopene.

**Figure S2 – Effect of bixin (A) and lycopene (B) on cell viability of macrophage determined by MTT assay.**

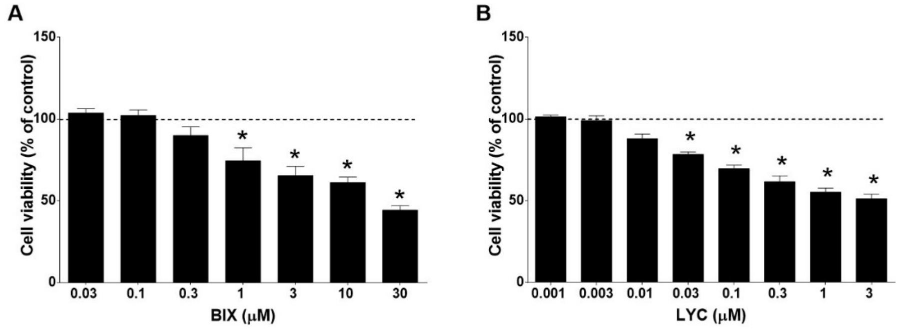


Each bar represents the mean ± SEM of at least three independent experiments. *Significantly different from control ($p<0.05$). BIX: bixin; LYC: lycopene.
